# Tubercle Bacilli in Cardiac Vegetations

**Published:** 1908-07-04

**Authors:** 


### Pathology.

### TUBERCLE BACILLI IN CARDIAC VEGETATIONS.

It is a remarkable fact that both the heart and the pericardium are affected macroscopically by the tubercle bacillus upon only the very rarest occasions. Why this should be so is one of the mysteries of pathology. The tubercle bacillus can affect the pleura, causing plastic adhesions or serous effusion; it can affect the peritoneum in similar ways, causing either ascites, or fibrous matting together of all the abdominal contents; but it affects the pericardium very seldom indeed. The pneumococcus affects the pleura quite often, the pericardium fairly often, and the peritoneum upon a smaller number of occasions. In rheumatic fever the pericardium is attacked more often that the pleuræ are, whilst the peritoneum is relatively immune. Why these differences should exist in the behaviour of the allied serous cavities to different micro-organisms it is hard to say. It can scarcely be a matter of anatomy alone. More likely the grounds for the differences are rather chemical than structural.

It is rare even to find a grey tubercle in the heart when, in cases of general tuberculosis, the lungs and pleuræ, the liver, spleen, kidneys, and meninges, are crammed with miliary tubercles. Notwithstanding this, there are well-marked exceptions to this rule, and it occasionally happens that tubercle bacilli find a habitat upon the lining of the heart or upon the cardiac valves, and cause a fungating or malignant endocarditis. The following is an instance of this uncommon condition:

The patient, a worker in the fields, twenty-six years of age, suffered from chronic mania, alternating with melancholia, and had to be sent to an asylum. She was taken there on March 10, 1905, having been in perfect health hitherto, except in regard to her mental state. In May 1906, during a period of extreme depression, she began to have pyrexia, and at the same time physical signs suggestive of phthisis were detected in the lungs. Tubercle bacilli were found in the sputum; in short, the patient died of tuberculosis on June 6, 1907.

At the autopsy, the upper lobe of the left lung was riddled with cavities, and the rest of the lungs was full of caseous broncho-pneumonic areas; the left kidney contained two foci of crude tubercle, each the size of a pea. The heart exhibited the most interesting lesions of all: its right side was natural; but the left ventricle presented upon its anterior wall, near the apex, a mass of grey vegetations as big as a hazel nut, but irregular in outline. The mitral and aortic valves were not involved.

Microscopically, the vegetations consisted of fibrin in which were embedded red and white corpuscles, and many epithelioid cells. There were no giant cells nor giant-celled systems, but the Ziehl-Neelsen method of staining brought out tubercle bacilli, which were much more numerous near the surface than deeper in.

The first impression was that the condition was one of tuberculous endocarditis; the next, that possibly there had been an ordinary terminal endocarditis of peculiar position, to the surface of which some of the tubercle bacilli circulating in the blood had become mechanically adherent. To decide between these two possibilities would have been impossible in this case, were it not that a good deal of experimental work has been done upon this subject in animals. After having been for a long time in doubt, these tuberculous lesions of the endocardium seem now to be accepted without dispute. They were formerly attributed to secondary infections; and doubtless they sometimes are such; but observers such as Lion, Cornil, Thirg, and Etienne have all demonstrated tubercle bacilli in the vegetations; and experiments upon animals in the hands of Michaelin, Blum, and others have clearly proved the fact that Koch's bacillus is able to produce inflammatory lesions of the endocardium. These observers, after injuring the aortic valves of the rabbit, and injecting tubercle bacilli intravenously, demonstrated the production of warty endocarditis, with soft fungating masses on the injured valves.

It is a remarkable fact that fungating endocarditis due to tubercle bacilli presents precisely the same appearances as does fungating endocarditis due to other micro-organisms. The two are indistinguishable macroscopically. Moreover, it is not sufficient merely to make ordinary microscopical sections for the detection of the tubercle, for no giant-celled systems are formed in the vegetations as they are in living tissues. To detect the tuberculous nature of the endocarditis, it is necessary to use the special bacteriological methods of staining for the bacilli.

Bernard and Salomon have brought forward a new and interesting experimental confirmation of this fact. With very skilful technique they injected an emulsion of tubercle bacilli directly into the left ventricles of two dogs and six rabbits. The animals were killed in from twenty to fifty days after the operation. Four of the rabbits and one of the dogs exhibited cardiac lesions. The latter consisted in granulomata beneath the pericardium, in the myocardium, and upon the endocardium. The structure of the lesions of the pericardium and myocardium exhibited typical giant-celled systems; but the vegetations upon the aortic valves not only looked for all the world like those of ordinary infective endocarditis, but also, in histological preparations stained in the usual way, exhibited no giant cells or other changes by which tubercle could be diagnosed. It was only by staining the sections by the Ziehl-Neelsen method that the tuberculous nature of the vegetations was shown by the occurrence in them of tubercle bacilli arranged precisely as in the vegetations from the heart of the case we have described.

From a pathological point of view this seems to be a very important fact to be aware of; for acute infective endocarditis in persons suffering from consumption might easily be regarded as resulting from secondary infection if macroscopic evidence alone were relied upon in distinguishing tuberculous from non-tuberculous endocarditis.

